# Boerhaave’s syndrome: the role of conventional chest X-ray

**DOI:** 10.1590/0100-3984.2018.0087

**Published:** 2018

**Authors:** Claudio Marcio Amaral de Oliveira Lima, Waldyr Maymone, Tatiana Mendonça Fazecas

**Affiliations:** 1 Hospital Municipal Jesus, Departamento de Radiologia, Rio de Janeiro, RJ, Brazil.

*Dear Editor,*

It was with great enthusiasm that we read the article “Boerhaave’s syndrome: a differential diagnosis of chest and abdominal pain” published in the March/April 2018 issue of Radiologia Brasileira^(1)^. Although the article mentioned that the use of conventional imaging methods is of great value in the immediate detection of esophageal rupture, we would like to add some information to the text based on simple X-rays, given that the article provided only computed tomography images.

**Figure 1:**
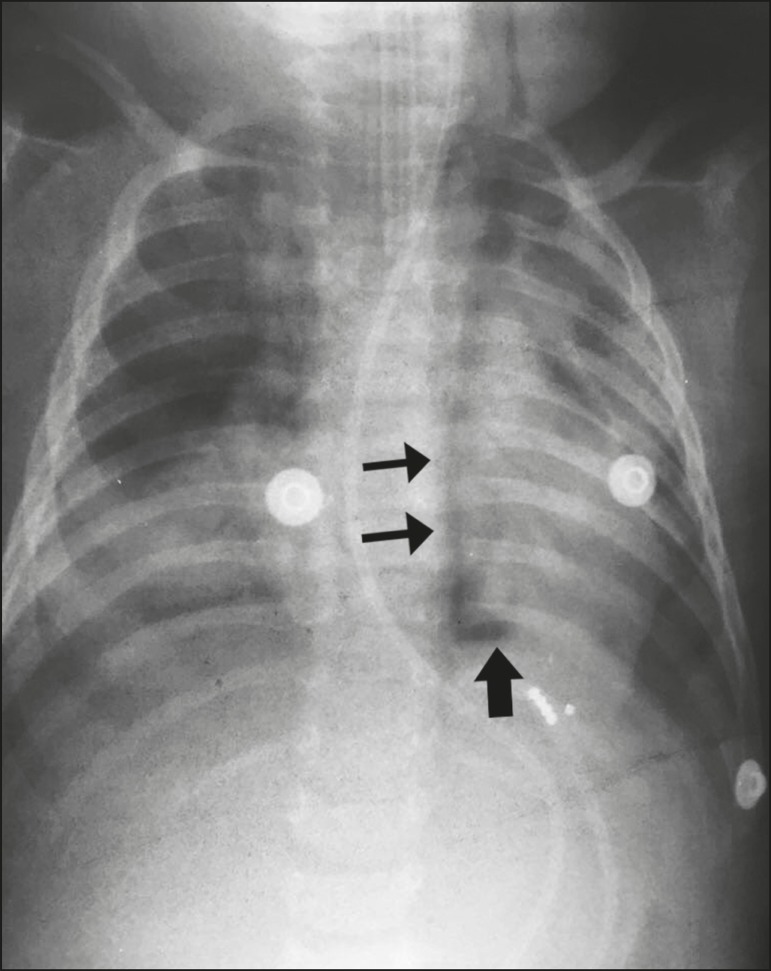
The V sign of Naclerio. A one-year-old male, hospitalized with a diagnosis of pneumonia in the lower left lobe, with no satisfactory response to treatment. After the insertion of a nasogastric tube, there was worsening of the clinical status, a chest X-ray showing the V sign of Naclerio and suggesting a diagnosis of esophageal rupture with pneumoperitoneum. Vertical branch (thin arrows) and horizontal branch (thick arrow).

In spontaneous esophageal rupture (Boerhaave’s syndrome), the diagnostic radiological finding is the V sign of Naclerio (Figure 1), identified on a chest X-ray as two hypertransparent V-shaped lines, one along the left border of the aorta and the other creating the continuous diaphragm sign on the left. The sign is produced by the presence of air between the left diaphragm and the descending aorta (vertical branch of the V) and between the left diaphragm and the parietal pleura (horizontal oblique branch of the V). The V sign was first described in 1957 by a thoracic surgeon, Emil A. Naclerio (1915-1985), in patients with rupture in the left posterolateral region of the esophagus^(2)^. However, the sign is not pathognomonic and might not be seen in (iatrogenic or traumatic) lesions at the level of the proximal esophagus^(2-4)^.

Bladergroen et al.^(5)^ observed that esophageal lesions were iatrogenic, secondary to endoscopy, in up to 55% of cases; spontaneous in 15%; caused by a foreign body in 14%; and due to trauma in 10%. Other chest X-ray findings that indicate pneumoperitoneum include pneumopericardium, the continuous diaphragm sign, the continuous left hemidiaphragm sign, the V sign of brachiocephalic vein confluence, and the ring-around-the-aorta sign^(2-4)^. Simple X-ray is a useful, practical, fast, and portable method that can be employed in severely ill patients hospitalized in closed units, which makes it a very important diagnostic tool, long used and still of great utility, commonly being the only imaging resource available; therefore, it is critical that radiologists know how to identify Boerhaave’s syndrome and other serious diseases from the X-ray findings^(2-4)^. We acknowledge the importance of computed tomography in assessing thoracic conditions. However, we would like to emphasize that the clinical profile, together with the chest X-ray findings, is usually sufficient to diagnose pneumomediastinum^(2)^.
